# Juvenile primary Fibromyalgia Syndrome: epidemiology, etiology, pathogenesis, clinical manifestations and diagnosis

**DOI:** 10.1186/s12969-021-00493-6

**Published:** 2021-03-01

**Authors:** Maya Levy Coles, Rotem Weissmann, Yosef Uziel

**Affiliations:** 1 Pediatric Rheumatology Unit, Department of Pediatrics, Meir Medical Center, 49 Tshernichovsky St., 44281 Kfar Saba, Israel; 2 Sackler School of Medicine, Tel Aviv University, Tel Aviv-Yafo, Israel

**Keywords:** Fibromyalgia, Juvenile, JPFS, Chronic pain, Musculoskeletal pain syndrome

## Abstract

Juvenile primary fibromyalgia syndrome (JPFS) is a chronic, musculoskeletal pain syndrome affecting children and adolescents, most commonly adolescent girls. The syndrome has a multifactorial etiology, with altered central pain processing playing an important role. The hallmark symptom is severe, widespread musculoskeletal pain. Other symptoms include sleep and mood disturbances, headaches, stiffness, and subjective joint swelling. Physical examination can reveal multiple tender points. The diagnosis is clinical, with defined criteria. Early diagnosis and intervention are important. In this part of the review, we discuss the epidemiology, etiology, pathogenesis, clinical manifestations and diagnosis of JPFS. Part two will focus on treatment and prognosis.

## Background

Chronic musculoskeletal pain is one of the main reasons for referrals to pediatric rheumatologists [1]. It can be caused by a wide variety of inflammatory and non-inflammatory conditions, including arthritis, hypermobility, fibromyalgia (FM), growing pains and complex regional pain syndrome (CRPS). Amplified musculoskeletal pain (AMP) syndrome is a generic, descriptive term used to describe chronic pain syndromes of undetermined etiology, such as FM, CRPS and idiopathic musculoskeletal pain. For individuals with AMP, pain signals are intensified; thus, mildly painful or nonpainful stimuli are registered by the body as very painful. This leads to attempts to avoid pain, leading to functional disability.

In our previous review, we focused on CRPS [2]. In this review, we focus on juvenile primary fibromyalgia syndrome (JPFS), also referred to as fibrositis, diffuse amplified pain or chronic widespread pain. Yunus and Masi were the first to describe and use the term JPFS in a 1985 clinical study. They suggested diagnostic criteria based on 33 juveniles, ages 17 years or younger, who suffered from chronic pain [3].

JPFS is defined by chronic, diffuse, musculoskeletal aching and pain— the hallmarks of this condition, as well as multiple, predictable tender points (TP), which are characteristic sites reported as painful to digital pressure. Part one of this review summarizes the current information regarding the epidemiology, etiology, clinical manifestations and diagnosis of JPFS. Part two focuses on its treatment and prognosis.

## Epidemiology

The worldwide prevalence of FM is 2.7%; ranging from 0.4 to 9.3% depending on the geographic location [4]. The estimated prevalence of JPFS ranges from 1.2 to 6.2% [5–9].

JPFS is more common among girls than boys [3, 6–8, 10–12]. Most cases of JPFS are white [3, 7, 10–12]. The mean age of onset is approximately 11.4 to 13.7 years, ranging from 5 to 18 years [3, 6, 7, 10]. The mean age at diagnosis is about 14.5 to 15.5 years, ranging from 7 to 18 years [3, 10, 11]. JPFS has been reported in younger children, as well. Eraso et al. found that among children younger than 10 years of age, the mean onset of JPFS was 7.5 years and the mean age at diagnosis was 10 years [11]. It is presumed that JPFS in younger children is underdiagnosed, and that symptoms are attributed to other causes [11].

### Etiology and pathogenesis

Fibromyalgia is considered a multifactorial disorder. There are many hypotheses regarding the causes of FM in adults, including enhanced pain perception, dysregulation of neuroendocrine axes, microtrauma to muscles, joint hypermobility (JH), poor physical fitness, disordered mood and disordered sleep [13]. Twin studies in adults suggest genetic, as well environmental influences [14]. However, studies of the pathogenesis of JPFS are sparse and it cannot be assumed that the pathogenesis and etiology of JPFS is like that of adult-onset FM.

#### Altered pain processing

Research suggests that central sensitization, a phenomenon of hyperexcitability of central nervous system (CNS) nociceptive circuits, has a role in the pathophysiology of JPFS [15]. Changes in CNS synaptic transmission (in receptors, neurotransmitters, ion channels and signaling pathways) due to genetic disposition and extrinsic factors, increase sensitization to an extent that results in the perception of non-nociceptive stimuli as painful, as well as exaggerated (prolonged and widespread) pain perception of noxious stimuli [16]. Studies have reported an imbalance between excitatory and inhibitory neurotransmitter levels in brain regions related to pain and sensory processing, such as the insula [17]. Studies using functional magnetic resonance imaging among individuals with FM have shown changes in the degree of connectivity between brain regions involved in pain perception that are associated with decreased pressure-pain thresholds [18], as well as higher sensitivity to pain provocation and impaired pain inhibition [19]. In a study of adult patients with FM, functional magnetic resonance imaging and machine-learning techniques that analyzed responses to painful pressure and nonpainful stimuli were used to identify a brain-based FM signature. Combined patterns identified FM patients vs. healthy controls with up to 92% sensitivity and 94% specificity. The altered responses observed in FM patients correlated with clinical pain, depression and disability [20].

Evidence of decreased mu-opioid receptor availability and of increased levels of endogenous opioids in the cerebrospinal fluid of individuals with FM, suggest a paradoxical effect of the endogenous opioid system, which is generally considered antinociceptive. This may explain the lack of efficacy of opioid analgesics in FM, and the possible role of opioid activity blockers, such as naltrexone, in the treatment of FM.

In addition to the prominent role CNS factors seem to play in pain amplification, peripheral nociceptive input may also contribute to the pathogenesis of FM. Patients with FM have impaired small nerve fiber function and morphology compared with healthy controls. This has been demonstrated in adult studies using objective testing methods, such as pain-related evoked potentials, quantitative sensory evaluations and examination of skin biopsies using electron microscopy [21, 22]. A meta-analysis indicates a 49% prevalence of small nerve fiber pathology in adult patients with FM [23] and recent evidence suggests that this may be related to symptom severity [24]. Aberrant expression of systemic and cutaneous microRNAs (which regulate molecular factors determining nerve de- and re-generation) were found to correlate with reduced density of small nerve fibers in patients with FM [25].

Some studies demonstrated abnormalities in the hypothalamic-pituitary-adrenal axis and autonomic nervous systems in individuals with FM [26, 27]; yet, further studies are needed to confirm these findings.

In summary, the amplified pain characteristics of FM may be related to CNS hyper-excitability and decreased pressure-pain thresholds.

#### Genetics and epigenetics

Familial aggregation of FM cases is well-documented [28–30]. A study by Schanberg et al. of 29 parents of children with JPFS, found that 79% of parents reported a personal history of at least one chronic pain condition, most commonly leg/foot pain, shoulder/neck pain or low back pain [31]. A Finnish twin cohort study of FM estimated heritability of approximately 50% [32]. These findings lead to the assumption that there is a genetic basis for the development of FM. Some studies have suggested a possible connection between human leukocyte antigens (HLA), especially HLA-DR4 and FM [33]. However, others have reported no association between specific HLA alleles and the development of FM [34]. Studies of genetic markers related to various neurotransmitters involved in pain processing found associations between FM and certain genetic polymorphisms influencing the serotonergic, dopaminergic and catecholaminergic pathways, in addition to other potential candidate genes [35–37].

Gene-environmental interactions have been theorized as a trigger for the development of FM, through epigenetic changes. The activation or inactivation of epigenetic pathways can become enhanced when cells experience rapid environmental changes, such as those due to injury and inflammation, resulting in hyperalgesia (distortion of pain perception) [38]. For example, studies have demonstrated a link between FM and hypomethylated patterns of genes associated with DNA repair, autonomic system response, and subcortical neuronal abnormalities [36].

In summary, gene-environmental interactions might be a factor in the pathogenesis of FM.

#### FM and the microbiome

A recent novel study comparing the microbiomes of 77 women with FM to 79 controls without FM, demonstrated a distinct pattern in the fecal microbiome of FM [39]. They found a quantitative association between the abundance of several types of bacterial strains and the severity of FM-related symptoms, such as pain intensity, pain distribution, fatigue, sleep disturbances and cognitive symptoms. FM patients were found to have lower abundance of *Faecalibacterium prausnitzii*, *Bacteroides uniformis*, *Prevotella copri* and *Blautia faecis*, bacteria, all of which have been demonstrated as depleted in patients with intestinal, inflammatory and rheumatoid conditions. The metabolic activity of some of these species might be relevant to the expression of FM symptoms. Possible mechanisms include short-chain-fatty-acids, bile acids, neurotransmitters and bacterial antigens in the host circulation [40]. Additional studies of the microbiome of FM patients are needed to further understand its pathogenesis, assist in its diagnosis and develop new therapeutic modalities.

#### Inflammation

Unlike rheumatologic diseases with a classic inflammatory nature, such as arthritis or vasculitis, in pain syndromes including FM, inflammation is not a significant etiologic factor. However, inflammation may play a role in the pathogenesis of FM. Theories suggest that dysregulation of immune-inflammatory pathways promotes alterations in the brain circuitry that modulates responses to pain and mood [41]. Inflammatory mechanisms in the peripheral tissues, spinal cord and brain, involving neuropeptides, chemokines and cytokines, lead to activation of the innate and adaptive immune systems among patients with FM. This is hypothesized to result in clinical symptoms such as swelling and dysesthesia, and to affect central symptoms, such as fatigue and changes in cognition [42]. Pain severity and other FM symptoms may be correlated with serum levels of TNFα and IL-8 [43]. Some studies even suggest that there is a subtype of FM (referred to as inflammatory FM) that can be identified by abnormal levels of various inflammatory markers [44].

In summary - inflammation is probably not a key factor in the pathogenesis.

#### Psychological and social factors

Psychological disorders may play a part in the etiology of FM. Studies show a significant association between FM and various psychological or psychiatric disorders, such as anxiety and depression [45, 46]. These associations may result from shared pathophysiology (such as alterations in neurotransmitter systems) [47] or common environmental triggers (such as stress or trauma). Traumatic events may precede the onset of FM. Elevated rates of posttraumatic stress disorder in children and adults with FM, and a history of childhood trauma, such as physical, sexual or emotional abuse, or neglect, have been demonstrated [48, 49]. A case-control study reported that the association between depression and FM may be attributable to certain types of childhood maltreatment [50]. While the role of psychological disorders in the etiology of FM is unclear, they are associated with greater symptom severity and importantly, often complicating FM treatment. For example, anxiety disorder in JPFS patients is associated with poorer functioning [45], and the diagnosis of a current major mood disorder is associated with greater physical impairment [51]. FM patients with posttraumatic stress disorder exhibit increased severe pain and fatigue, and poorer health-related quality of life [52]. Among JPFS patients, a history of trauma may be associated with greater psychological impairment [53].

In summary, psychological and social factors play an important role in the etiology and severity of FM.

#### Sleep disturbances

Sleep disturbances are common in FM patients, not only because of pain, but as part of the pathogenesis of the disease. Sleep dysfunction and other FM symptoms may be due to a shared pathophysiology, in that neurotransmitters that affect pain transmission also affect sleep [47]. A disrupted melatonin circadian rhythm, demonstrated in women with FM, as compared to healthy controls, was associated with poorer sleep quality, greater susceptibility to pain, a larger number of trigger points on physical evaluation, and higher rates of depressive symptoms [54].

Studies among healthy adult populations indicate that sleep dysfunction affects pain tolerance, and consequently may be presumed to be a risk-factor for FM symptoms. Polysomnography and psychophysical pain assessments showed that sleep disturbance may impair pain-inhibition and increase spontaneous pain [55]. Epidemiological studies indicate that poor sleep quality is a risk-factor for the development of myalgia, tenderness, and chronic widespread pain [56, 57].

The role of sleep in children and adolescents with JPFS has been assessed by objective sleep measures, such as actigraphy, polysomnography and sleep latency tests [58–60]. Based on these measures, JPFS was associated with greater sleep-onset latency, more awakenings, decreased total sleep time and sleep efficiency, more periodic limb movements, and more alpha-delta slow-wave sleep, as compared to healthy controls. There is no consensus as to whether the finding of increased alpha-delta sleep is connected to chronic pain or sleep anomalies [58, 59].

In juvenile FM patients, sleep disturbances increase functional disability, and thus, are an important factor in the pathogenesis of the condition, and may complicate its treatment [61].

#### Hypermobility

Joint hypermobility has been strongly linked to FM in studies of children and adults [62–64]. One hypothesis is that JH may cause recurrent microtrauma and occasional joint dislocation, leading to recurrent, localized joint pain, which may eventually cause central sensitization and progression to FM [63, 65]. JH syndrome, a common manifestation of dysautonomia, is more common in FM patients than in controls [66]. JH and dysautonomia are relatively common in juvenile FM patients, as compared with adults, possibly because ligamentous laxity declines with age [67]. In a study of 131 adolescent FM patients, JH was associated with significantly greater pain sensitivity, with lower TP thresholds and a greater number of painful TPs [64]; supporting the role of JH in the pain experience of patients with JPFS.

#### Growing pains

A connection between growing pains (GP) and the future development of FM has been hypothesized. GP are the most common cause of recurring musculoskeletal pain in children [68], and children with GP have more TP and lower pain thresholds than controls do [69]. A 5-year follow-up study by Uziel et al. of 35 children with GP, found that none developed FM, although the small study cohort was a major limitation to the detection of FM, which is far less common than GP [70].

#### Environmental factors

Among adults, FM may be triggered by various environmental “stressors”, such as physical trauma [71] and infections [72], but more evidence is needed to support these associations. An internet survey of over 2500 adult patients with FM found that among those who associated a specific event with their symptom onset, 26.7% described an acute illness. In addition, 43% of patients reported worsening symptoms during infections [73]. No evidence was found regarding the possible efficacy of antibiotic or anti-viral treatment in the management of FM. A possible role of vaccinations in precipitating FM has not been established.

## Clinical manifestations of JPFS

The characteristic symptom of JPFS is widespread musculoskeletal pain, usually with a very high subjective severity score [3, 74–76] . In adolescents with JPFS, pain has a negative effect on well-being and is associated with functional impairment, commonly leading to avoidance of regular daily activities [77, 78], school absenteeism [79], and poor social functioning [1, 80]. Pain severity, as well as measures of function and well-being, are significantly worse in youth with JPFS, as compared to those with other pediatric rheumatic diseases, as was shown in a study of 7753 patients enrolled in a multinational registry [81]. Functional impairment was significantly greater in males [76].

Sleep disturbances are another common manifestation of JPFS, including general fatigue, poor sleep, and feeling tired and unrefreshed upon waking in the morning [3, 10–12]. In a study of 201 JPFS patients enrolled in a multi-site patient registry, fatigue and disordered sleep were reported by 84 and 82% of patients, respectively [76]. Sleep disorders can have a direct negative impact on FM patients’ quality of life. Reid et al. found an association between disordered sleep and increased functional disability among children with JPFS [61].

Patients with FM may have various psychiatric comorbidities. Mikkelson et al. found that children with JPFS had significantly higher scores on the Children’s Depression Inventory than did children with widespread pain, without JPFS [46].

It is important to acknowledge that FM patients are not a homogeneous group, and not all patients exhibit psychiatric symptoms. For example, Giesecke et al. identified a subgroup of adult FM patients who exhibited extreme pain tenderness but lacked any associated psychological or cognitive factors [82]. Another study found that nearly 28% of FM patients had healthy or low affective balance style, related to less functional disability and psychiatric comorbidity, suggesting the presence of an emotionally resilient subgroup [83].

Headache, a common symptom, was reported in 68% of JPFS patients [76].

Additional symptoms in JPFS patients include stiffness, subjective joint swelling, and abdominal pain [10–12, 84]. These symptoms may be more common in children with disease onset prior to the age of ten years, as shown in a study of 148 JPFS patients by Eraso et al. [11]. Chronic chest pain in adolescents may be another manifestation of JPFS [85].

## The diagnosis and evaluation of JPFS

Diagnosis of JPFS is clinical, based on a detailed history and physical examination, including a thorough neurological assessment. Other than possible findings of tender points or increased pain susceptibility to touch, no positive neurological signs, such as change in tonus, strength or sensorial sensation should be found. Patients with JPFS often undergo assessments by their primary pediatrician and multiple specialists prior to their eventual diagnosis [86]. This is because JPFS is under-recognized by healthcare providers, which can lead to delays in diagnosis from 6 months to as long as 5 years, and thus, to a delay in proper treatment, which increases the negative impact on patients’ lives (mood, functionality, academic achievement, etc.) [84, 86, 87]. Additionally, lack of recognition of JPFS increases the medical costs for the patient, their families and the healthcare system, due to many office visits, medications and diagnostic tests [86].

JPFS is considered part of the spectrum of functional pain syndromes or central sensitivity syndromes [15, 88, 89]. Consequently, the symptoms of JPFS frequently overlap those of other functional pain syndromes, such as irritable bowel syndrome, chronic fatigue syndrome, temporomandibular joint disorder, myofascial pain syndromes, premenstrual syndrome, tension-type headaches and mood and anxiety disorders [88–92]. Therefore, as part of the diagnostic process, other causes of chronic pain should be considered and ruled out (Table 1), including chronic idiopathic musculoskeletal pain that does not result from FM [1, 93]. Other medical conditions should also be considered and ruled out. An example is Familial Mediterranean Fever which can be presented with exertional leg pain [94].

**Table 1:** Differential diagnosis of pediatric chronic musculoskeletal pain

Diagnosis	Distinguishing characteristics
Complex regional pain syndrome	Localized chronic musculoskeletal limb pain that may be accompanied by allodynia, hyperalgesia, swelling and/or changes in skin color of the affected limb; dry, mottled skin; hyperhidrosis and trophic changes of the nails and hair.
Hypermobility	Common, younger ages (preschool to elementary school); pain more severe toward the end of the day, usually associated with specific activities; evidence of hypermobility on physical examination.
Myofascial pain	Pain arises from sustained contraction of a muscle, especially in the head, jaw or upper back. Presence of a trigger point (tender point) and reproduction of the pain by maneuvers that place stress on proximal structures or nerve roots.
Unrecognized local pathology (fracture, strain, sprain)	Trauma/strain to the affected limb; pain worsens with physical activity and excursive; positive findings in plain radiographs.
Arthritis	Inflammation of one or more joints; pain is constant and localized to the affected joint; positive physical findings.
Spondyloarthropathy	Lumbar spinal pain associated with arthritis. Imaging or other evidence of arthritis affecting the sacroiliac joints and the lumbar spine; response to nonsteroidal anti-inflammatory drugs.
Leukemia	Child appears sick; anorexia and lethargy are present; fever is common; nocturnal pain and bone pain. Abnormal blood count, relative thrombocytopenia, and elevated erythrocyte sedimentation rate.
Spinal cord tumors	Slow progression of pain; pain quality is low with steady intensity; abnormal neurologic examination; pathologic magnetic resonance imaging.
Chronic nonbacterial osteomyelitis	Chronic, noninfectious inflammation in the metaphyses close to the physes of multiple bones. Bony tenderness over the affected sites. Presence of lytic lesions on plain radiographs. Lesions appear on bone scan. Pain usually responds to nonsteroidal anti-inflammatory drugs or corticosteroids.
Raynaud’s disease	Cold or emotional stress causes vasospasms which induce discoloration of the fingers, toes, and occasionally other areas. Episodes are short-lived. Pain, numbness or tingling can be experienced with the episode. Pain can be reproduced with a cold challenge. Digital tip ulcers might occur.
Fabry disease	Deficiency of ceramide trihexoside α-galactosidase, X-linked recessive inheritance. Episodic excruciating burning pain in the hands and feet. Symptoms usually begin in adolescence. Presence of bluish maculopapular hyperkeratotic lesions around the perineum, elevated erythrocyte sedimentation rate.
Erythromelalgia	Rare disorder characterized by burning pain, warmth, and redness of the extremities. Can be familial or secondary to myeloproliferative disorders. Pain alleviated by cold exposure.
Pernio	Episodic inflammatory skin condition, presenting after exposure to cold as pruritic and/or painful erythematous-to-violaceous acral lesions, recurs with cold exposure.
Chronic compartment syndrome	Usually occurs in athletes. Repetitive loading or exertional activities cause exercise-induced pain that is relieved by rest. Onset of symptoms typically occurs at a specific exercise distance, interval or intensity level. Symptoms tend to subside with rest and are minimal during normal daily activities.
Peripheral mononeuropathy	More common among adults. Occurs following injury or infection. Can cause severe burning pain in the distribution of the involved peripheral nerve. Findings on physical examination are limited to the area supplied by the injured nerve.
Progressive diaphyseal dysplasia	Begins in adolescence. Causes severe leg pain, fatigue, headaches, weight loss, weakness, abnormal waddling gait. Diagnosis confirmed via plain radiographs, which demonstrate cortical thickening and sclerosis of the diaphysis of the long bones.
Idiopathic juvenile osteoporosis	Uncommon. Typically occurs just before the onset of puberty, involving pain in the lower back, hips, and feet, often accompanied by difficulty walking. Fractures of the lower extremities can occur. Plain radiographs demonstrate low bone density, fractures of weight-bearing bones, and collapsed or misshapen vertebrae. Bone scans can demonstrate microfractures.
Thyroid disease	Hyperthyroidism/hypothyroidism can cause widespread musculoskeletal pain. History and physical examination reveal signs and symptoms of thyroid disease. Abnormal thyroid function test.
Vitamin D deficiency	Uncommon in developed countries. Causes limb pain. Low levels of vitamin D in laboratory tests.
Juvenile idiopathic arthritis	Joint swelling, morning stiffness, elevated inflammatory markers, elevated anti-nuclear antibodies.
Systemic lupus erythematosus	Rash, systemic inflammation, elevated inflammation markers, decreased complement, and elevated specific antibodies, including anti-nuclear antibodies.
Myositis and myopathies	May occur prior to a recent infection; weakness, elevated muscle enzymes.

### History

As the diagnosis of JPFS is clinical, a thorough history is very important. The history should address pain properties, typically revealing widespread musculoskeletal pain with high subjective pain severity. The presence and severity of additional symptoms, including sleep disturbances, psychological comorbidities and other somatic manifestations should be mapped. The degree of functional impairment should be assessed, including school absenteeism, and avoidance of regular daily activities and peer relationships. Measures of well-being should be evaluated. It is imperative to include information regarding the child’s familial, social and academic environments in the primary assessment, due to their importance in treatment.

### Physical examination

The physical examination of children with FM might reveal multiple TP and in some cases evidence of JH, but it is otherwise unimpressive, with no evidence of arthritis or other pathological findings [9–12, 63].

The presence of multiple TP in the physical examination of adult and pediatric FM patients, as compared to their healthy peers reflects their generalized sensitivity to pain [3]. In a study of 47 female adolescents with JPFS, the mean number of TP was 11, compared to an average of 2–3 in healthy controls [75]. Children with FM may have fewer TP, as compared to adults. Children and adolescents with FM were found to have a mean of 9.7 TPs summed over all visits; fewer than the criterion of 11 established for adults at a single visit [12].

Use of the manual TP examination as a diagnostic criterion is controversial, as it is frequently performed incorrectly. The amount of pressure applied is inconsistent, as well as the degree of subjectivity and lack of reproducibility between examiners and over time. In addition, it is less applicable for men because they generally appreciate smaller amounts of pain from pressure [74, 95].

King et al. quantitatively assessed pressure pain thresholds in 34 adolescent females with JPFS, using a hand-held algometer. Participants with JPFS had significantly higher pressure pain sensitivity than did healthy controls [96]. Therefore, it has been suggested that a manual TP examination may not be necessary in the diagnosis of JPFS.

During physical examination, JPFS patients often demonstrate a significant difference between their reported high pain intensity and the minimal pain behaviors they demonstrate. This is in addition to an ill-matched affect (complaints of intense pain while remaining calm and happy) [97, 98].

### Further evaluation

Often, after a thorough history and physical examination, the diagnosis of JPFS is quite clear, requiring no further evaluation. However, when the diagnosis is uncertain or when another pain diagnosis is suspected (Table 1), bloodwork and imaging are often evaluated. Baseline laboratory tests include a complete blood count, blood chemistry, C-reactive protein, erythrocyte sedimentation rate, and creatinine kinase. If anamnesis suggests systemic constitutional symptoms such as lupus, an antinuclear antibody (ANA) should be obtained. Most FM patients have normal laboratory values [3, 11, 97], although some have a low positive ANA titer. However, this is found incidentally in approximately 5% of the general population and is inconsequential when there is no clinical evidence of inflammatory or autoimmune disease [11, 99]. Imaging studies are usually normal, with the exception of disuse osteoporosis, that might be present if immobility is prolonged [100].

### Diagnostic criteria for JPFS

Yunus and Masi were the first to propose criteria for JPFS, in 1985 (Table 2) [3]. Although they have never been validated, to date they are the only criteria specifically developed for diagnosing FM in the pediatric population and are commonly used by pediatric rheumatologists. Other sets of criteria were developed by the American College of Rheumatology (ACR) for the diagnosis of FM in adults. Although the 1990 ACR criteria [101] were used to evaluate JPFS, they were never validated in the pediatric population. In 2010, the ACR suggested new criteria for the clinical diagnosis of FM in adults [102]. These criteria were evaluated for use in the diagnosis of JPFS in adolescent females, with the Yunus and Masi criteria as the gold standard, and found to have a sensitivity of 89.4% and specificity of 87.5%, suggesting that they can be applied to this population [75]. Notable advantages of the 2010 ACR criteria over the Yunus and Masi or the 1990 ACR criteria, include assessment of symptom severity and of additional somatic FM symptoms, ease and rapidity of use, and elimination of the TP examination (which has been criticized due to its subjectivity and inconsistent administration and results) [74, 95]. The ACR adult criteria were revised in 2010/2011 and in 2016 [103, 104], but they have not been evaluated for use in the pediatric FM population.

**Table 2:** Yunus and Masi suggested diagnostic criteria for Juvenile Primary Fibromyalgia Syndrome [3]

Major criteria	
1) Generalized musculoskeletal aching at 3 or more sites for 3 or more months	
2) Absence of an underlying condition/cause	
3) Normal laboratory tests	
4) Five or more typical tender points^a^	
Minor criteria	
1) Chronic anxiety or tension	
2) Fatigue	
3) Poor sleep	
4) Chronic headaches	
5) Irritable bowel syndrome	
6) Subjective soft tissue swelling	
7) Numbness	
8) Pain modulated by physical activities	
9) Pain modulated by changes in weather	
10) Pain modulated by anxiety/stress	
JPFS is diagnosed when all major criteria are met, plus three of the minor criteria or when there are four tender points and five minor criteria.	

^a^Yunus and Masi listed 31 tender points. However, after the 1990 American College of Rheumatology criteria were published, most rheumatologists use the 18 tender points suggested there [101]: occiput, low cervical, trapezius, supraspinatus, second costochondral junction, lateral epicondyles, gluteal folds, posterior greater trochanter and the medial knee fat pad

Most recently, in 2018, the American Pain Society with the U.S. Food and Drug Administration and the Analgesic, Anesthetic, and Addiction Clinical Trial Translations Innovations Opportunities and Networks developed and published a new set of criteria for diagnosing FM in adults [105]. However, they have yet to be validated, and their application to pediatric FM cases has not been studied.

To date, there are no gold standard criteria for the diagnosis of JPFS, and no consensus among pediatric rheumatologists as to which of the existing criteria should be used. Furthermore, the diagnosis of JPFS is fluid, as symptoms may change or evolve over time. Thus, children with diffuse idiopathic pain should be reassessed periodically by their rheumatologist.

### Clinical measures of FM

Assessment of symptoms, including pain, fatigue, sleep, functioning, and quality of life, is primarily based on patient report [106]. Measures that can be used to assess juvenile FM, include the Functional Disability Inventory, the Modified Fibromyalgia Impact Questionnaire – Child Version, and the Pediatric Quality of Life 3.0 Rheumatology Module Pain and Hurt Scale [107].

## Conclusions

Juvenile primary fibromyalgia syndrome is a chronic, musculoskeletal pain syndrome. It is more common among adolescent girls. The etiology is multifactorial, with genetic and environmental influences. Central pain sensitization which causes amplification of pain signals is an important contributing mechanism. The hallmark symptom of JPFS is diffuse widespread musculoskeletal pain, usually with a high subjective severity score. Patients often suffer from functional impairment and reduced well-being, causing avoidance of regular daily activities. Other symptoms include sleep disturbances, mood disturbances, headaches, stiffness, subjective joint swelling, abdominal pain, and manifestations of dysautonomia. Physical examination can reveal multiple predictable TP; yet, this finding is less prominent in children than in adults and is controversial as a diagnostic criterion. JH is sometimes observed. Diagnosis is clinical, based on a detailed patient history and thorough physical examination. Further evaluation is usually unnecessary. Other causes of chronic pain must be considered and ruled out. Since the Yunus and Masi criteria for diagnosis of JPFS were developed in 1985, no other criteria have been specifically designed for diagnosing FM in the pediatric population. However, of the 2010 ACR criteria developed for adult FM has been evaluated for use in adolescents, with satisfactory results [75].

## Data Availability

Data sharing is not applicable to this article as no datasets were generated or analyzed.

## Abbreviations

ACR: American College of Rheumatology
AMP: Amplified musculoskeletal pain
ANA: Anti-nuclear antibody
CNS: Central nervous system
CRPS: Complex regional pain syndrome
DNA: Deoxyribonucleic acid
FM: Fibromyalgia
GP: Growing pains
HLA: Human leukocyte antigens
JH: Joint hypermobility
JPFS: Juvenile primary fibromyalgia syndrome
TP: Tender point
